# Topical treatment with fresh human milk versus emollient on atopic eczema spots in young children: a small, randomized, split body, controlled, blinded pilot study

**DOI:** 10.1186/s12895-015-0027-9

**Published:** 2015-05-04

**Authors:** Teresa Løvold Berents, Jørgen Rønnevig, Elisabeth Søyland, Peter Gaustad, Gro Nylander, Beate Fossum Løland

**Affiliations:** Institute of Clinical Medicine, University of Oslo, Oslo, Norway; Department of Dermatology, Oslo University Hospital, Oslo, Norway; Department of Research, Education and Innovation, Oslo University Hospital, Oslo, Norway; Department of Microbiology, Oslo University Hospital, Oslo, Norway; Norwegian National Advisory Unit on Breastfeeding, Womens and Children´s Division, Oslo University Hospital, Oslo, Norway

**Keywords:** Atopic eczema, Children, Human milk, Emollient, Topical treatment, Pilot study

## Abstract

**Background:**

Public health nurses report on effects of fresh human milk as treatment for conjunctivitis, rhinitis and atopic eczema (AE), the latter being highly prevalent in early childhood. Emollients and topical corticosteroids are first line treatment of AE. As many caregivers have steroid phobia, alternative treatment options for mild AE are of interest. The aim of this small pilot study was to assess the potential effects and risks of applying fresh human milk locally on eczema spots in children with AE.

**Methods:**

This was a split body, controlled, randomized and physician blinded pilot study, of children with AE with two similar contralateral eczema spots having a mother breastfeeding the child or a sibling. Fresh expressed milk and emollient was applied on the intervention spot and emollient alone on the control area, three times a day for four weeks. The severity and area of the eczema spots was evaluated weekly, and samples from milk and the spots were analysed weekly with respect to bacterial colonisation.

**Results:**

Of nine patients included, six completed the study. Mean age at inclusion was 18.5 months. The spots examined were localized on the arms, legs or cheeks. The spots were similar in severity, but differed in area. In one patient the eczema ceased after inclusion. In four patients both control and intervention areas increased during the intervention. The relative change in eczema area compared to baseline showed less increase in the intervention spots in two patients, whereas the opposite was observed in three. In four children *Staphylococcus aureus* was found in their eczema once or more. In three of the 28 human milk samples, *Staphylococcus aureus*, *alfa haemolytic streptococci* or *coagulase negative staphylococci* were detected. *Staphylococcus aureus* was found once both in human milk and in the eczema spots, no clinical signs of infection were however observed. No secondary infection due to milk application was detected.

**Conclusion:**

In this small pilot study, no effect was found on eczema spots treated with topical application of fresh human milk. (ClinicalTrials.gov Identifier, NCT02381028).

## Background

Atopic eczema (AE) is a common, chronic, pruritic, relapsing skin disease which affects up to 20% of children in the Nordic European countries [1]. AE is strongly associated with other atopic disorders, such as allergic rhinitis and asthma [2]. The pathogenesis is interplay between barrier dysfunction, genetic, immunological, environmental factors and colonization by *Staphylococcus aureus* (*S. aureus*) [3].

The treatment algorithm in AE is based on treating the barrier defect, the inflammation, the infection and the pruritus [4]. First line treatment is treating the barrier defect with optimal skin care by the use of emollients and baths. If a clinical infection is present in the eczema, local antiseptics may be utilized; for severe cases systemic antibiotics are needed. The inflammation is treated with topical steroid creams. Topical steroids have been shown to be a well-tolerated treatment, but in spite of this many caregivers have steroid phobia, mainly because of the potential side effects [5]. A treatment option in chronic eczema is topical calcineurin inhibitors; these are however not to be used in children under two years of age [4]. Alternatives without side effects for young children are therefore of interest.

Human milk may represent a source with potential treatment properties. Knowledge of the immunological qualities of mammalian milk can be traced back to 1892, when Paul Ehrlich demonstrated that newborn mice were protected against the toxic effects of phytotoxins if they were fed milk from an immunized mouse [6]. Today, numerous studies have contributed to our present knowledge of the short- and long-term effects of human milk in the breastfed child [7]. Mammalian milk is species specific. Human milk contains specialized immune components, including factors with anti-microbial and anti-inflammatory properties [8], which theoretically could be responsible for an effect on eczema spots when applied topically.

In Norway, public health nurses report several cases where parents have had positive experiences with topic applications of expressed human milk in eyes of children with conjunctivitis and on eczema spots in children with AE. We have not been able to find any studies investigating such treatment in children with AE. However, local use of expressed human milk has been studied for diaper dermatitis, rhinitis and conjunctivitis [9,10].

The aim of this small pilot study was to assess the potential positive and/or negative effects of topical use of expressed, fresh human milk on eczema spots in young children with AE by evaluating the eczema areas. A secondary aim was to evaluate any bacterial transmission from human milk to the eczema spots, causing infection in the child. Finally the mothers’ compliance to the treatment was of interest.

## Methods

### Trial design

This was a split body controlled, randomized and physician blinded study of expressed human milk and emollients on contralateral eczema spots in children, the trial was registered at ClinicalTrials.gov Identifier, NCT02381028.

Inclusion criteria were children with AE according to Hanifin and Rajkas criteria [11] with a mother breastfeeding the child or a sibling. The eczema spots in the treatment and control areas were to be similar in features and extent as well as being localized on contralateral parts of the body. Children were excluded if the severity of the eczema spots indicated need for treatment with antibiotics and/or steroids.

The study was approved by the Regional Committee for Medical and Health Research Ethics - South East Norway. Mothers of participating children were informed verbally and in writing, and signed an informed consent prior to commencing the study.

### Recruitment

Study patients were recruited through advertisement posters from three different well baby clinics in Oslo, Norway, in the period 2008–2011. Mothers interested in the study contacted the study team. The consultations mainly took place at the hospital; a few were carried out in the child’s home. The mothers were able to contact the examining physician if they experienced any problems with the treatment.

### Intervention

The study intervention was local application of fresh human milk on the study area. By hand milking, the mothers were to squeeze out and throw away the first few droplets of milk, and then squeeze the next droplets directly from the nipple to the eczema spot. The number of milk droplets depended on the size of the eczema area; the mothers were instructed to cover the whole eczema spot with milk. After absorption of the milk droplets, both treatment and control areas were treated with moisturizing cream (Apobase creme®, Actavis Norway AS). The cream contains: Aqua, Paraffinum Liquidum, Petrolatum, Cetearyl Alcohol, Ceteareth-20, Ceteareth-12, Sodium Gluconate, Caprylyl Glycol, Phenoxyethanol, with a total lipid content of 30 percent. Both intervention and control areas were treated with this regimen three times a day for four weeks.

### Randomization and blinding

The same physician examined all the children, and was blinded as to which areas were the control or intervention sites. At inclusion the physician diagnosed the AE according to Hanifin and Rajkas criteria [11]. The two contra-lateral eczema spots to be randomized were elected; i.e. flexural aspect of elbow, flexural aspect of knees or cheeks. Another physician, who did not see the child, was responsible for the randomization. The child was given a randomization number and the mothers were then informed on which side to apply the fresh expressed human milk and emollient, and on which side to apply emollient alone.

### Follow-up

After inclusion, the children were examined once weekly for four consecutive weeks. The overall severity of AE was evaluated by the use of SCORAD, which defines mild disease as score <25, moderate disease between 25–50 and severe disease as scores >50 [12]. The severity of the study and control areas were evaluated by scoring the erythema, lichenification, excoriation and pruritus on a scale from 0 to 3, where 0 is none and 3 is severe. Study and control areas were measured using Visitrak™ (Smith & Nephew), a portable device used to measure the area of wounds [13]. A transparent folio is placed over the area and the borders are outlined, whereafter a computer determines the area measured in cm^2^. In the present study this device was used to follow the development of the extent of the eczema spots.

At each visit, samples for bacterial cultures were taken at eczema spots and from breastmilk, using Amies Agar Gel with Charcoal^TM^, Copan Venturi Transystem® (Copan, Brescia, Italy). Sampling from eczema spots was done by carefully rubbing the cotton swab over the eczema spot. Sampling of breastmilk was done from milk expressed by hand milking. The specimens were cultivated on blood agar plates and selective media for *S. aureus* and for gram-negative rods.

### Outcome

The primary outcome was to register proportional change in the area of the eczema spot from baseline, as measured by Visitrak™. The secondary outcome was to assess transmission of bacteria from mother’s milk to eczema spots in the child. The mother’s compliance was also evaluated.

### Statistical methods

Descriptive statistics were performed. The areas of the intervention and control sites for each child were not identical; therefore differences were calculated as percentages: Changes in the areas of the control and intervention sites each week were calculated as change in proportion of area related to baseline area.

## Results

### Study population

**Figure 1:**
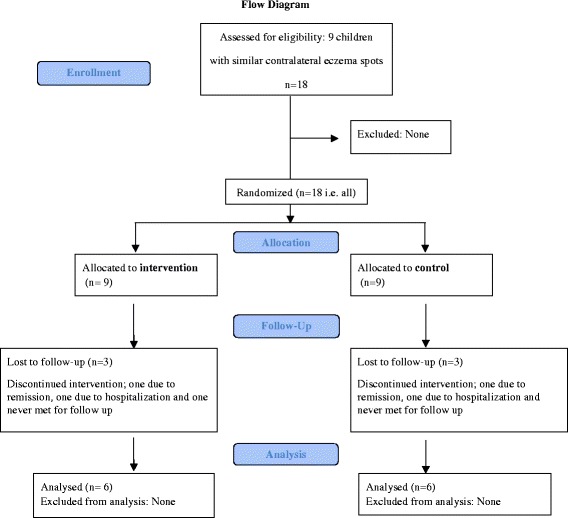
Flow diagram. Nine children with atopic eczema and bilateral eczema lesions were assessed for eligibility to a small split body, controlled, physician blinded pilot study evaluating human milk and emollient versus emollient alone on eczema lesions. Three children were lost to follow up.

Nine children, four male, were recruited for the study through advertisement posters from three different well baby clinics in Oslo, Norway in the period 2008–2011. Three of these nine children were lost to follow-up consultations; one experienced remission from AE, the second suffered from severe AE and was hospitalized, the third never met for follow up (Figure 1). Two children were treated with mothers’ milk produced for a younger sibling. The mean age of the children was 18.5 months (min, max; 4, 32). At inclusion mean SCORAD was 35 (min, max; 22, 45) and at the end of the study mean SCORAD was 34 (min, max; 18, 52). The spots examined were localized on the arms or legs in five of the children and on the cheeks in one. The spots were similar in severity, however the extent differed some.

### Changes in measured area of eczema

**Figure 2:**
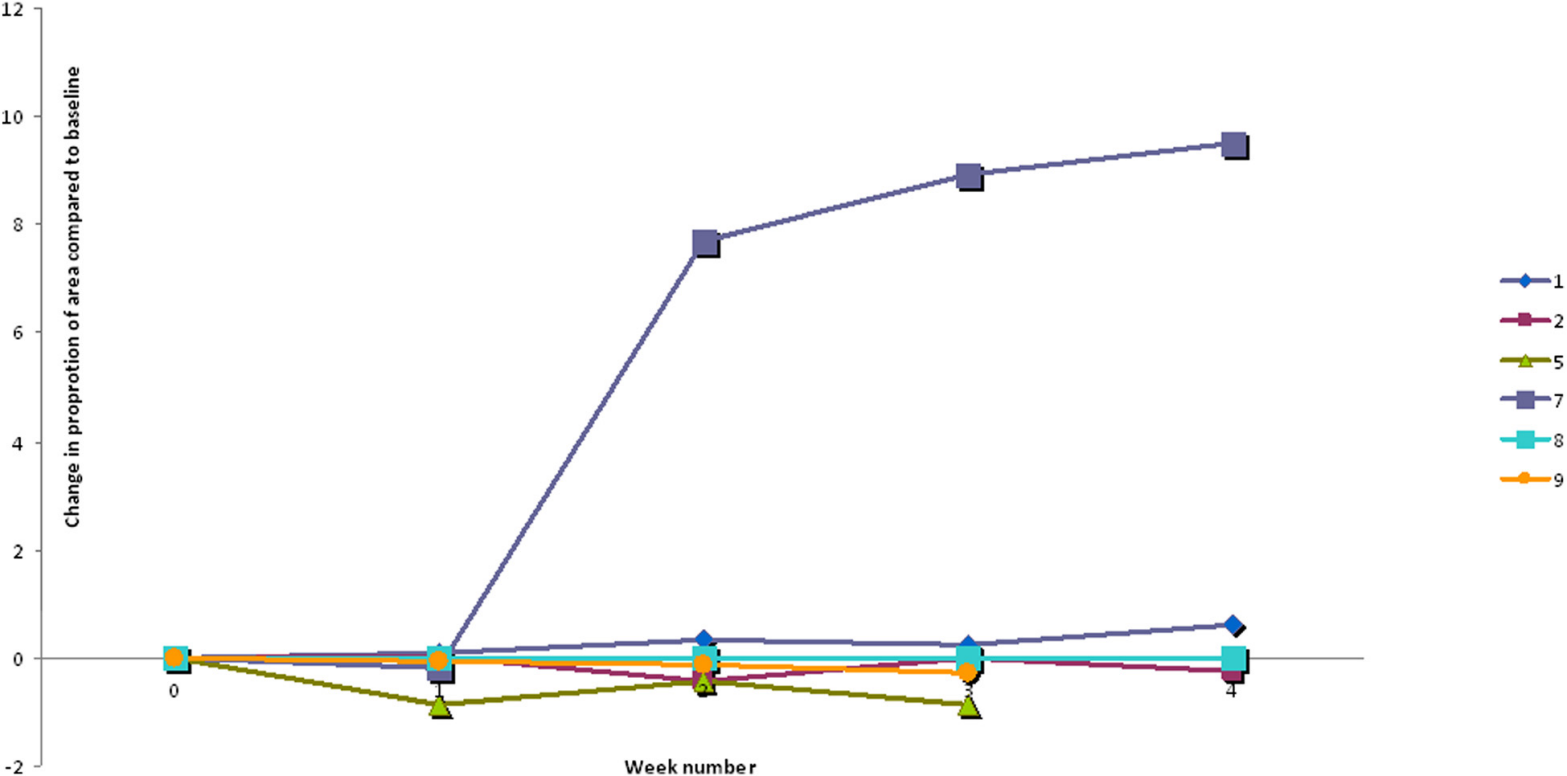
Change in eczema area. This figure illustrates the weekly difference between control and intervention sites based on the area change from baseline in six children with atopic eczema included in a split body, controlled, physician blinded pilot study evaluating human milk and emollient versus emollient alone on eczema lesions. Each line represents one child. The difference is calculated as: control area week 1, 2, 3 or 4 divided by control area at week 0 minus intervention area week 1, 2, 3 or 4 divided by intervention area at week 0. Lines above zero represent improvement of the intervention area, and lines below zero represent the relative increase of the eczema areas of the intervention sites.

The weekly change in the control and intervention eczema area related to baseline eczema area is illustrated in Figure 2. At the end of the study, child number one and seven displayed less area involvement in the area treated with human milk compared to the emollient treated area. In child number two, five and nine the emollient treated area showed at study end less involvement than the area treated with human milk. The eczema spots in child number eight disappeared after inclusion.

Most of the children showed an improvement of their general eczema, except for child five, who showed a slight increase. Child seven differs from the other children: this child experienced a worsening of the total eczema, having mild atopic eczema at inclusion, and severe atopic eczema at week four.

### Changes in presence of bacterial species

Four of the children had positive *S. aureus* cultures in their eczema once or more (Table 1). However, only in four of twelve occasions this coincided with clinical signs of infection. Gram-negative rods were found in child number one at one visit. *S. aureus, alfa haemolytic streptococci* or *coagulase-negative staphylococci* were detected in three of the 28 human milk samples. Only on one occasion the same bacteria (*S. aureus*) were detected in both the eczema lesions and the human milk (child number five), and signs of clinical infection were present (Table 1). The intervention areas differed some from the control areas, as *S. aureus* was found in intervention area but not in the control area on four occasions in three different children (Table 1).

**Table 1:** **Bacteria in eczema spots and human milk**

**Child**	**Week**	**Clinical infected**	**Study area**	**Control area**	**Human milk**
1	0	No	No growth	No growth	Skin flora^1^
	1	No	No growth	No growth	Missing
	2	No	No growth	Skin flora	Missing
	3	No	Gram negative rods	Gram negative rods	Skin flora
	4	No	Skin flora	No growth	Skin flora
2	0	No	Skin flora	Skin flora	Skin flora
	1	No	Skin flora	Skin flora	Skin flora
	2	No	Skin flora	Skin flora	Skin flora
	3	No	Skin flora	Skin flora	Skin flora
	4	No	No growth	No growth	Skin flora
5	0	No	*S. aureus* (rich)	*S. aureus* (mod.^2^)	Skin flora
	1	No	*S. aureus* (rich)	*S. aureus* (rich)	*S. aureus* (some col.^3^)
	2	Yes	*S. aureus* (mod.)	*S. aureus* (sparse)	Skin flora
	3	No	*S. aureus* (rich)	*S. aureus* (rich)	Skin flora
	4	Yes	*S. aureus* (mod.)	Skin flora	Skin flora
7	0	No	Skin flora	Skin flora	Skin flora
	1	Yes	Skin flora	Skin flora	CNS^4^
	2	No	*S. aureus* (some col.)	Skin flora	Skin flora
	3	No	Skin flora	Skin flora	Skin flora
	4	No	*S. aureus* (some col.)	Skin flora	Skin flora
8	0	No	Missing	Missing	Missing
	1	No	Skin flora	Skin flora	Skin flora
	2	No	Skin flora	Skin flora	Skin flora
	3	No	*S. aureus* (some col.)	Skin flora	Skin flora
	4	No	Skin flora	Skin flora	Skin flora
9	0	No	*S. aureus* (some col.)	*S. aureus* (sparse)	Skin flora
	1	Yes	*S. aureus* (mod.)	*S. aureus* (rich)	*AHST* ^*5*^
	2	No	*S. aureus* (sparse)	*S. aureus* (mod.)	No growth
	3	Yes	*S. aureus* (mod.)	*S. aureus* (some col.)	Skin flora

^1^Skin flora: non-pathogen bacteria belonging to the skin flora ^2^ mod.: moderate, ^3^col. colonies, ^4^CNS: coagulase negative staphylococci, ^5^AHST alfa haemolytic streptococci.

Bacterial presence in samples taken from eczema spots and human milk weekly, and the presence of clinically judged infection in control and intervention sites of six children with atopic eczema included in a split body, controlled, physician blinded pilot study evaluating human milk and emollient versus emollient alone on eczema lesions.

### Compliance

The mothers experienced the application of human milk as an uncomplicated treatment option.

## Discussion

In this small, split body controlled randomized pilot study of human milk and emollient applied topically on eczema spots in six children, no effect was found on eczema spots treated with the topical application of fresh human milk. In two of five children with persistent eczema lesions during the study, there was less involvement of the human milk treated area compared to the emollient area at study end compared to baseline. However, the opposite was found in three children.

There are few studies looking at the effect of human milk on eczema. One study of children with diaper dermatitis examined the effect of applying human milk after each breastfeeding or hydrocortisone 1% ointment twice a day, detecting after one week an effect of human milk comparable to that of hydrocortisone [9]. The application frequency was higher than in our study, but we still believe that an application rate of three times a day would be enough to show an effect of human milk, after four weeks of treatment.

There are many theoretical indications of how human milk can be effective on eczema lesions. One aspect of atopic disease is the type 2 helper T cell (Th2) dysbalance with production of interleukin- 4 (IL-4), IL-5, and IL-13 in the acute phase. In the chronic phase there is a Th1/Th0 dominance with production of interferon-γ, IL-2, IL-5 and granulocyte-macrophage colony-stimulating factor [2]. Of interest, therefore, are findings from an animal model, where the effect of human colostrum used locally on an acute inflammatory process was shown to be as potent as oral indomethacin and superior to oral dexamethasone at suppressing polymorphonuclear leukocyte influx [14,15]. In humans, the milk contains, among a wide variety of biologically active hormones, glucocorticoids. These are transferred from plasma to the milk in levels fairly highly correlated (in the .6-.7 range) [16].

Milk samples from healthy donors have revealed bacteria in 10–23% of the samples [17]. In the present study, bacteria were found in three of 28 samples; 11%. In one child only (child number five), *S. aureus* was found once in both the eczema spot and in the milk. Clinically, however, the eczema was not infected, and this child had *S. aureus* on both eczema lesions at every visit. This suggests that no iatrogenic infections due to application of fresh mothers milk occurred. Surprisingly, *S. aureus* was found in the intervention area but not in the control area on four occasions in three different children. One speculation might be that the preservative (eg. phenoxyethanol) in the emollient had some antimicrobial effect when applied alone.

Human milk contains several different substances that act against bacteria, virus and fungi, such as Secretory IgA and Secretory IgM [18], lactoferrin, lysozyme, oligosaccharides, Toll-like receptors and fatty acids [8]. In a study evaluating the inhibition in vitro of human colostrum against bacterial cultures from eye swabs of neonates with neonatal conjunctivitis, the inhibitory activity was ≥ 50% against *S. aureus* and coliform bacteria, demonstrating an antimicrobial effect also in vitro [19]. A consistent inhibitory effect of human milk was found against *Neisseria gonorrhea* in children with conjunctivitis. A significant but less pronounced effect was also found against *Moraxella catarrhalis* [10]. This strengthens the evidence for an antimicrobial effect of human milk also when used topically. When comparing different methods for preventing omphalitis in newborns, an application frequency of topical human milk twice a day demonstrated a shorter separation time of the umbilical stump compared to the use of antiseptics [20]. In the study of Ibhanesebhor [19], the mean duration of inhibition of human milk against *S. aureus* was three hours. Considering the huge amount of different biologically active components and cells in human milk, there might be a variety of candidates for explaining the positive effects described above.

The children participating in the present study were recruited through advertisement posters in well baby clinics, and responding mothers were presumably highly motivated and inclined to trust alternative/new treatment methods. We cannot rule out that the mothers co-treated the intervention sites with for instance steroid cream, but the instructions with respect to treatment of the intervention and control site were clear, and the results do not indicate performance bias.

## Conclusions

The results of this small randomized, controlled pilot study of six children with AE does not support an effect of topical applied human milk. Treatment with fresh expressed human milk seems safe and easy for mothers to carry out.
